# Metagenome-Assembled Genomes of Bacterial Symbionts Associated with Insecticide-Resistant and -Susceptible Individuals of the Glassy-Winged Sharpshooter (Homalodisca vitripennis)

**DOI:** 10.1128/mra.00506-22

**Published:** 2022-06-16

**Authors:** Cassandra L. Ettinger, Frank J. Byrne, Richard A. Redak, Jason E. Stajich

**Affiliations:** a Department of Microbiology and Plant Pathology, University of California, Riverside, Riverside, California, USA; b Department of Entomology, University of California, Riverside, Riverside, California, USA; c Institute for Integrative Genome Biology, University of California, Riverside, Riverside, California, USA; University of Southern California

## Abstract

The role of microbes in insecticide resistance is an emerging question. Here, we describe six metagenome-assembled genomes (MAGs) associated with the glassy-winged sharpshooter (Homalodisca vitripennis [Germar, 1821]) (Hemiptera, Cicadellidae). MAGs representing the obligate symbionts *Candidatus* Sulcia muelleri and *Candidatus* Baumannia cicadellinicola and the facultative symbiont *Wolbachia* were obtained from imidacloprid-resistant and imidacloprid-susceptible sharpshooters.

## ANNOUNCEMENT

The glassy-winged sharpshooter (Homalodisca vitripennis) is an invasive xylem-feeding leafhopper that is native to the southeastern United States and Mexico and was recently introduced into California (1, 2). Insecticide-based management of invasive H. vitripennis populations to control for disease transmission has led to high levels of imidacloprid and pyrethroid resistance (3). Recent studies in other insects have proposed a role for microbes in the resistance mechanisms of hosts (4–8). Here, we present metagenome-assembled genomes (MAGs) to help explore the role of microbes in the resistance of the glass-winged sharpshooter.

Sharpshooters were collected from California citrus groves in Tulare and Kern counties in August 2019 (3). DNA was extracted from an imidacloprid-susceptible sharpshooter (A6) from Tulare county and from an imidacloprid-resistant individual (C9) from Kern county following the 10× Genomics protocol for high-molecular-weight genomic DNA extraction from single insects (9, 10). DNA libraries were made by the University of California, Riverside, Institute for Integrative Genome Biology (IIGB) Genomics Core using a NEBNext Ultra II DNA library preparation kit and underwent 150-bp paired-end sequencing on an Illumina NovaSeq 6000 system at the Vincent J. Coates Genomics Sequencing Laboratory at the University of California, Berkeley, to produce 322.3 million and 223.8 million reads associated with the A6 and C9 libraries, respectively.

We used the anvi’o v.7 workflow to generate MAGs associated with the resistant and susceptible sharpshooters (11). FastQC was first used to confirm high read quality and the absence of adapters (12). Because read quality was high across the entire read length (Q scores of >Q20), no additional trimming was performed prior to assembly. Next, we separately assembled the reads for A6 and C9 using SPAdes v.3.15.2 (13). For each assembly, we computed the sequencing coverage by aligning the reads with Bowtie2 v.2.4.2 and SAMtools v.1.11 (14, 15). We used anvi-gen-contigs-database to make databases for each assembly and predicted open reading frames with Prodigal v.2.6.3 (16). Single-copy bacterial genes (17) were identified using HMMER v.3.2.1 (18), and rRNA genes were identified using barrnap (19). Taxonomy was predicted for genes using Kaiju v.1.7.2 (20) with the NCBI BLAST nonredundant protein database, including fungi and microbial eukaryotes, v.2020-05-25. We constructed anvi’o profiles using contigs of >2500 bp via anvi-profile and anvi-merge. Contigs were clustered into MAGs using CONCOCT v.1.1.0 and MetaBAT2 v.2.12.1 (21, 22). We ran DASTOOL (23) to obtain an optimized set of MAGs, which were then manually assessed using anvi-interactive and anvi-refine. MAG completeness and redundancy were calculated using the CheckM v.1.1.3 lineage-specific workflow (24), and MAGs were assigned taxonomy using GTDB-Tk v.1.3.0 (25). We used D-GENIES to align MAGs to reference genomes (*Candidatus* Sulcia muelleri [GenBank assembly accession number GCA_000017525.1] and *Candidatus* Baumannia cicadellinicola [GenBank assembly accession number GCA_000013185.1]) with Minimap2, given that these obligate symbionts have reduced genomes (26–29).

We obtained three MAGs representing known symbionts of H. vitripennis for each of the resistant and susceptible sharpshooters (Table 1), including obligate “*Ca.* Sulcia muelleri,” obligate “*Ca*. Baumannia cicadellinicola,” and facultative *Wolbachia* sp. (28–31). Given that these symbionts provide important biological functions for their hosts, we used the anvi’o pangenomic workflow (32) and anvi-compute-functional-enrichment to assess whether functions were enriched in symbionts based on host insecticide resistance status, but we found no enriched functions (33). Future work is needed to assess whether there exist population variations in symbiont MAGs correlated with host insecticide resistance status and whether nonsymbiont microbiome members have roles in the resistance mechanisms of H. vitripennis.

**TABLE 1:** Genomic summary of MAGs from insecticide-resistant and -susceptible glassy-winged sharpshooters

Bin identifier^*a*^	Putative taxonomy	Genome size (bp)	No. of contigs	*N*_50_ (bp)	No. of genes^*b*^	GC content (%)	Coverage (×)	CheckM completion (%)	CheckM contamination (%)	Anvi'o completion (%)	Anvi'o contamination (%)	Reference alignment (%)^*c*^	GenBank accession no.
RES-01	“*Ca.* Sulcia muelleri”	214,450	33	8,141	227	21.96	2,659.66	35.7	0.1	59.15	0	86.55	JALIDL010000000
SUS-01	“*Ca.* Sulcia muelleri”	242,982	8	62,435	232	22.33	2,297.96	41.98	0	66.20	0	98.49	JALIDI010000000
RES-02	“*Ca.* Baumannia cicadellinicola”	661,039	60	13,771	636	33.45	3,557.67	95.93	1.25	90.14	0	95.70	JALIDM010000000
SUS-02	“*Ca.* Baumannia cicadellinicola”	676,761	9	116,162	612	33.08	2,133.69	100	0	92.96	0	98.50	JALIDJ010000000
RES-03	*Wolbachia* sp.	1,410,832	121	17,062	1,333	33.95	186.75	98.08	1.28	90.14	0	NA	JALIDN010000000
SUS-03	*Wolbachia* sp.	1,392,537	122	16,150	1,314	33.98	100.21	98.08	1.28	90.14	0	NA	JALIDK010000000

a RES, resistant; SUS, susceptible.

b No. of genes predicted by Prodigal.

c Percent alignment of obligate symbiont MAGs to respective reference genomes by D-GENIES using Minimap2. NA, not applicable.

### Data availability.

Sequence reads for imidacloprid-susceptible (A6) and imidacloprid-resistant (C9) H. vitripennis individuals were deposited under BioProject accession numbers PRJNA717305 and PRJNA819061, respectively. The raw sequence reads were deposited under SRA accession number SRR14269173 for A6 and under SRA accession number SRR18455411 for C9. The MAG assemblies have been deposited in DDBJ/ENA/GenBank under the accession numbers JALIDI000000000, JALIDJ000000000, JALIDK000000000, JALIDL000000000, JALIDM000000000, and JALIDN000000000. The versions described in this paper have accession numbers JALIDI010000000, JALIDJ010000000, JALIDK010000000, JALIDL010000000, JALIDM010000000, and JALIDN010000000. MAGs were annotated after deposition by the NCBI Prokaryotic Genome Annotation Pipeline (PGAP) (34). Related computational scripts for this work are available on GitHub and archived in Zenodo (https://doi.org/10.5281/zenodo.6493649) (35).
